# Localization of Capsule Endoscope in Alimentary Tract by Computer-Aided Analysis of Endoscopic Images

**DOI:** 10.3390/s25030746

**Published:** 2025-01-26

**Authors:** Ruiyao Zhang, Boyuan Peng, Yiyang Liu, Xinkai Liu, Jie Huang, Kohei Suzuki, Yuki Nakajima, Daiki Nemoto, Kazutomo Togashi, Xin Zhu

**Affiliations:** 1 Graduate Department of Computer Science and Engineering, The University of Aizu, Aizuwakamatsu 965-8580, Japan; d8232118@u-aizu.ac.jp (R.Z.);; 2 Division of Coloproctology, Aizu Medical Center, Fukushima Medical University, Aizuwakamatsu 969-3492, Japan; 3 Department of AI Technology Development, M&D Data Science Center, Institute of Integrated Research, Institute of Science Tokyo, Chiyoda, Tokyo 101-0062, Japan

**Keywords:** capsule endoscopy, deep learning, computer-aided analysis, transformer

## Abstract

Capsule endoscopy is a common method for detecting digestive diseases. The location of a capsule endoscope should be constantly monitored through a visual inspection of the endoscopic images by medical staff to confirm the examination’s progress. In this study, we proposed a computer-aided analysis (CADx) method for the localization of a capsule endoscope. At first, a classifier based on a Swin Transformer was proposed to classify each frame of the capsule endoscopy videos into images of the stomach, small intestine, and large intestine, respectively. Then, a K-means algorithm was used to correct outliers in the classification results. Finally, a localization algorithm was proposed to determine the position of the capsule endoscope in the alimentary tract. The proposed method was developed and validated using videos of 204 consecutive cases. The proposed CADx, based on a Swin Transformer, showed a precision of 93.46%, 97.28%, and 98.68% for the classification of endoscopic images recorded in the stomach, small intestine, and large intestine, respectively. Compared with the landmarks identified by endoscopists, the proposed method demonstrated an average transition time error of 16.2 s to locate the intersection of the stomach and small intestine, as well as 13.5 s to locate that of the small intestine and the large intestine, based on the 20 validation videos with an average length of 3261.8 s. The proposed method accurately localizes the capsule endoscope in the alimentary tract and may replace the laborious real-time visual inspection in capsule endoscopic examinations.

## 1. Introduction

Despite the high prevalence of alimentary tract diseases, small intestinal diseases are difficult to visualize using conventional diagnostic modalities, e.g., radiography. Alternative endoscopic techniques such as push enteroscopy, ileocolonoscopy, and intraoperative enteroscopy have been developed; however, these procedures are invasive and distressing for the patients [1]. Capsule endoscopy has established itself as a minimally invasive, low-risk tool that offers a comprehensive visualization of the entire gastrointestinal tract with advanced sensors [2,3].

Capsule endoscopy offers essential advantages for the inspection of the gastrointestinal tract, as it allows for access to regions that are difficult to visualize with conventional endoscopy [4]. The accurate localization of specific gastrointestinal intersections in capsule endoscopy videos is critical for endoscopists to confirm the progress of capsule endoscopic examinations. In clinical practice, the location of a capsule endoscope should be constantly monitored through a visual inspection of the endoscopic images by medical staff to confirm the examination’s progress. The automatic localization of a capsule endoscope remains challenging due to the unpredictable movement of the capsule within the gastrointestinal tract. Most of the current localization methods rely on external devices such as antenna arrays despite non-real-time localization [5]. Therefore, an automated and unconstrained localization solution is crucial for improving the overall efficiency of capsule endoscopy inspection [6].

Recent advancements in AI have increasingly contributed to capsule endoscopy analysis, demonstrating its high accuracy and reliability for medical image analysis. For example, Li et al. proposed a video segmentation method based on color features, achieving an average accuracy of 91.2% for stomach/small intestine classification and 89.2% for small intestine/large intestine classification [7]. Wang et al. developed a classification algorithm for the stomach and small intestine, reaching a sensitivity of 99.7% by introducing a classifier based on the color–texture fusion feature of visual perception [8]. Srivastava et al. proposed a high-speed intestinal image classification model named FocalConvNet. For the Kvasir-Capsule dataset, the model achieved a weighted F1-score of 0.6734. Additionally, it demonstrated a throughput of over 148 images per second on a Tesla V100 GPU [9]. Bai et al. proposed a classification algorithm based on the Vision Transformer, which achieved an accuracy of 79.15% on the multi-class classification tasks of the Hyper-Kvasir capsule endoscopy disease dataset [10]. Ganesh et al. proposed a model that combines a convolutional neural network (CNN) with a Vision Transformer. The model first uses the CNN as a feature extractor and then feeds the extracted features into the Vision Transformer for further classification. The model achieved a classification accuracy of 94% on the Kvasir-V1 dataset [11]. Xiao et al. proposed a lesion detection algorithm for gastrointestinal diseases based on YOLOv8 as the backbone network and integrated with the Swin Transformer module. In the detection task including 23 classes of lesions, the algorithm achieved an mAP50 of over 91%, with an mAP50 exceeding 99.4% for 11 classes of lesions [12]. Son et al. proposed an organ intersection localization algorithm based on ResNet for spatial feature extraction and temporal filtering for noise reduction [13]. Their algorithm achieved a transition time error of 70.85 s. Chung et al. proposed a visualization system for gastrointestinal organ classification and transitional areas based on a code-free platform [14].

Despite the progress that AI has made in the analysis of capsule endoscopy, to the best of our knowledge, few studies have focused on the automatic localization of a capsule endoscope. The development of an automated localization system without external devices would not only reduce the constraint required for inspection but also increase the overall efficiency of gastrointestinal inspections.

The goal of this research is to propose a novel capsule endoscope localization system to address the limitations of the existing approaches. The main contributions of this paper are as follows:

1. Evaluating the performance of classification models in capsule endoscope images: This study systematically evaluated the classification performance of multiple classification models for capsule endoscope images;

2. Proposing a localization system based on inspection videos: This method locates the position of the capsule endoscope in the alimentary tract only using the complete inspection video, without requiring any additional equipment;

3. Demonstrating the adaptability of the localization system: The system’s performance was evaluated by integrating it with different classification models, demonstrating its strong adaptability across various models.

In this paper, Section 2 (Materials and Methods) describes the details of the dataset used in this study, the architecture of the proposed localization system, and relevant hyperparameter settings. Section 3 (Results) presents the experimental results and the performance of the proposed system. Section 4 (Discussion) includes the discussion on the advantages and the limitations of the proposed method. Finally, Section 5 (Conclusion) concludes the paper with a summary of the findings.

## 2. Materials and Methods

### 2.1. Data

**Figure 1:**
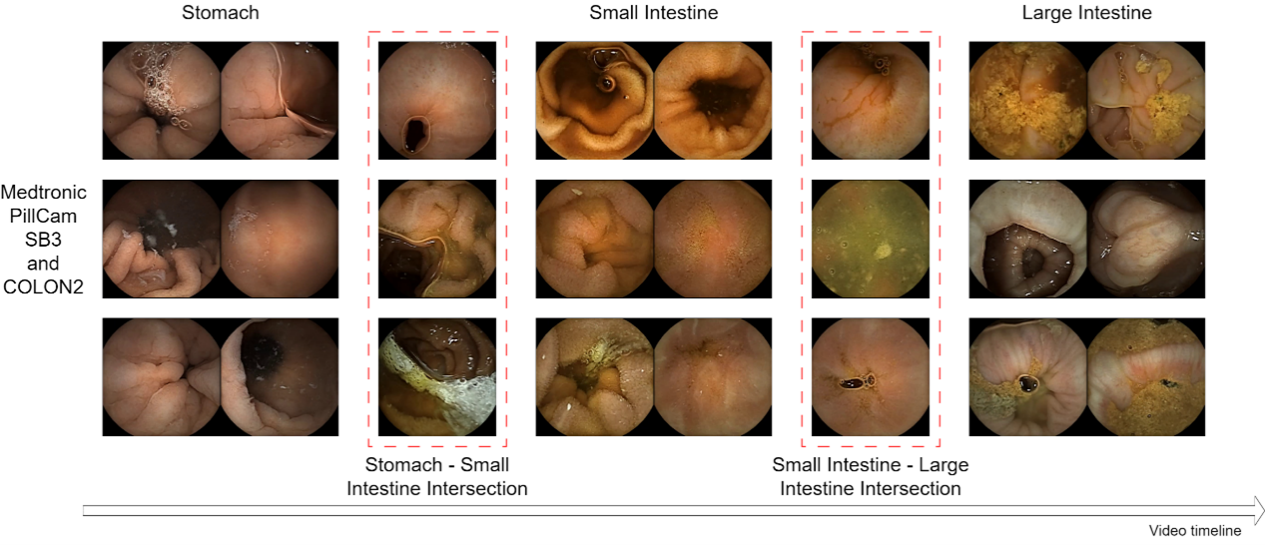
Frames from three capsule endoscopy videos. The intersections between the stomach and small intestine and between the small intestine and colon are indicated by a red dashed line.

In this study, the capsule endoscopy videos of 204 cases recorded in 2014–2021 were consecutively collected from the Division of Proctology, Aizu Medical Center, Fukushima Medical University. The PillCam™ SB 3 Capsule or the COLON 2 System was used to perform capsule endoscopy. These systems included capsule endoscopes, sensor belts, sensor arrays, and recorders. The size of the individual frames in each video was 576 × 576. The frames per second (FPS) of the PillCam COLON 2 and the SB 3 capsule System were 4–35 and 2–6 FPS, respectively. This study was approved by the Institutional Review Board of Fukushima Medical University and was conducted in accordance with the relevant guidelines and regulations of the Declaration of Helsinki. Figure 1 shows the data used in this study, including the intersection points of the stomach to the small intestine and the small intestine to the large intestine. These intersection points were annotated by experienced endoscopists.

The endoscopists first prescreened all the data and removed eight incomplete and incorrect videos. Thus, 196 videos were used for the development. Two experienced endoscopists annotated all videos. The annotation included the following two landmarks: (1) the intersection of the stomach and the small intestine and (2) the intersection of the large and small intestines. Private patient information was removed from the videos for the training, validation, and testing. The frame size of all the videos was 576 × 576 pixels.

All cases were randomly grouped into 156 (80%), 20 (10%), and 20 (10%) cases for training, validation, and testing, respectively. The training set contained 13,734,139 images, of which 1,040,114, 11,153,742, and 1,540,283 were from the stomach, small intestine, and large intestine, respectively. The numbers of training images obtained from the stomach, small intestine, and large intestine were unbalanced. Therefore, the data in the training were sampled and set to 22,130 (1/47), 22,307(1/500), and 22,004 (1/70) images for the stomach, small intestine, and large intestine, respectively, to accelerate training and reduce the effects of imbalance in the data.

The validation set included 1,749,098 images, of which 161,203, 1,249,867, and 338,028 were obtained from the stomach, small intestine, and large intestine, respectively. The test set comprised 1,957,051 images, including 191,670, 1,550,400, and 214,981 images from the stomach, small intestine, and large intestine, respectively.

### 2.2. Methods

#### 2.2.1. Classification Method and Data Augmentation

The feasible backbone networks were selected from six pre-trained models—ResNet50, DenseNet121, Swin Transformer, Vision Transformer, VGG19, and Inception v4 [15,16,17,18]. Each model was pretrained using approximately one million images in ImageNet. ResNet50, DenseNet121, VGG19, and Inception v4 are CNNs, which mainly extract image features through convolution and pooling operations. In contrast, the Swin Transformer and Vision Transformer are models based on the attention mechanism [19,20]. Unlike CNNs, transformers can use the global information of an image and focus on larger receptive fields simultaneously [21]. These properties have led to their widespread application in medical image data [22].

**Figure 2:**
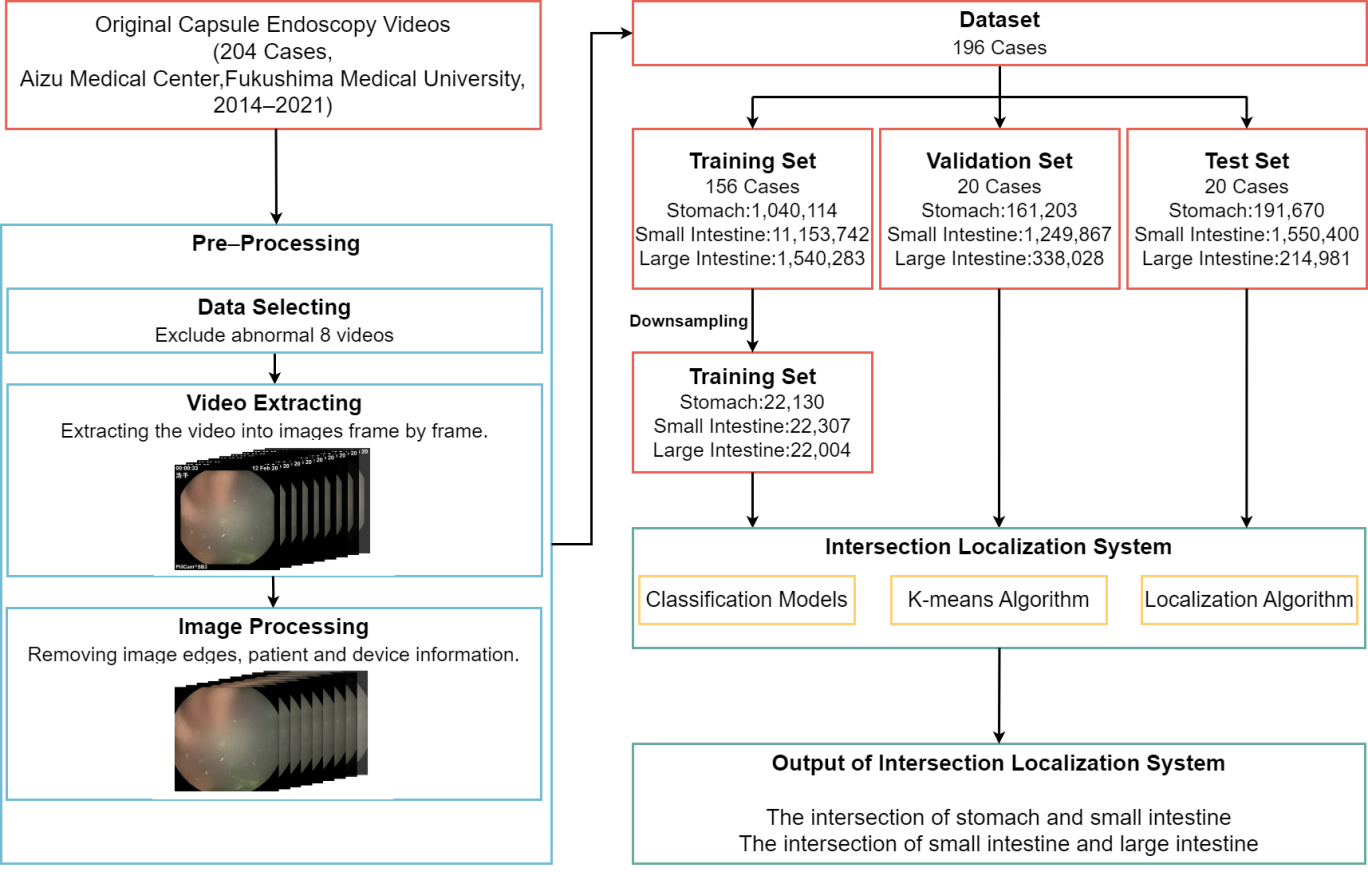
Flowchart of this study including data pre–processing, dataset division, and intersection localization.

Figure 2 illustrates the flowchart of this study. The main steps to achieve the primary objective are as follows: (a) A deep learning-based classification model was developed to predict the probability of each video frame belonging to the stomach, small intestine, or large intestine; (b) the composite predicted value (CPV) and K-means algorithms were applied to mitigate the noise from the errors of the classification model; (c) the proposed localization algorithm (OPLA) was used to identify the stomach–small intestine and small intestine–large intestine intersections using thresholding.

A mixup algorithm was used for data augmentation [23] as a state-of-the-art method for data enhancement [24]. Compared to the conventional data augmentation methods, mixup is effective in expanding the dataset in the case of a small dataset [25]. Related studies have shown that mixup can significantly improve the performance of image classification [26].

#### 2.2.2. Composite Predicted Value and K-Means Algorithm

Initially, the capsule endoscopy videos were extracted into frames. Each extracted frame was then processed using our trained classification model, which outputs a classification probability (CP) consisting of probabilities for the stomach, small intestine, and large intestine, and the corresponding predicted class labels. To improve the robustness of our classification results, a novel metric called the CPV is presented, which combines the classification probabilities of all categories into a single informative value. The purpose of the CPV is to reduce the dimensionality of the classification model output and to encode positional codes for the (1) stomach, (2) small intestine, and (3) large intestine. The computed CPV sequences were processed using a K-means clustering algorithm to detect potential outliers. Equation (1) demonstrates the calculation of the CPV as follows:

(1) $$CPV=\sum_{i=1}^{3}W_{i}*CP_{i},$$

where $W_{i}$ represents the encoded positional codes 1, 2, and 3 for the stomach, small intestine, and large intestine, respectively. $CP_{i}$ represents the classification probability for each class.

**Figure 3:**
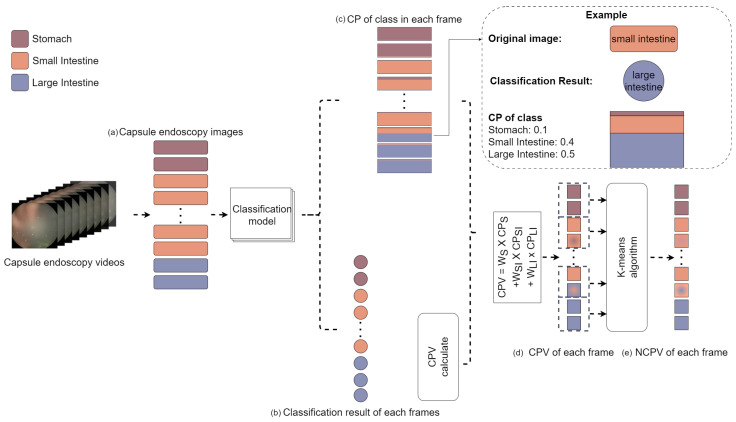
Visualization of the results of CPV and K-means algorithm in different gastrointestinal regions. (**a**,**b**) use colors to represent the stomach (brown), small intestine (orange), and large intestine (blue). (**c**) uses stacked bars to show CP values for each region per frame. (**d**,**e**) represent the CPV using a color gradient, where each square represents a frame and its color intensity correlates with the CPV magnitude, indicating the confidence of the model and the prediction of the predominant class.

Figure 3 presents the visualization results for the CPV and K-means algorithms. Figure 3a–e show the different visualization techniques to intuitively display the confidence and prediction results of the model in different gastrointestinal regions. Using color-coding schemes, stacked bar representations, and continuous color gradients, Figure 3 illustrates the performance of the algorithm for the stomach, small intestine, and large intestine. This visualization allowed for a quick understanding of the model’s prediction process and the distribution of results across the different gastrointestinal regions.

After computing the CPV for each video frame, a K-means clustering algorithm was applied for outlier detection. The K-means algorithm was chosen for its computational efficiency and effectiveness in partitioning data into different clusters [27]. In this study, the K-means algorithm refers to the K-means++ implementation in the scikit-learn library, which improved the original K-means by addressing issues such as sensitivity to initialization [28]. Additionally, we fixed the random seed to ensure the consistency and reproducibility of the model results. This step first identified the possible misclassifications and was critical for locating the intersections of the alimentary tract. K-means clustering was applied to sets of 200 consecutive frames, with a window size chosen to strike a balance between the spatial resolution and the computational efficiency. This method enables the detection of local anomalies while maintaining high sensitivity to gradual transitions between gastrointestinal regions.

#### 2.2.3. Localization Algorithm

In this step, a localization algorithm was proposed to localize the intersections between the stomach and the small intestine and between the small intestine and the large intestine.

**Figure 4:**
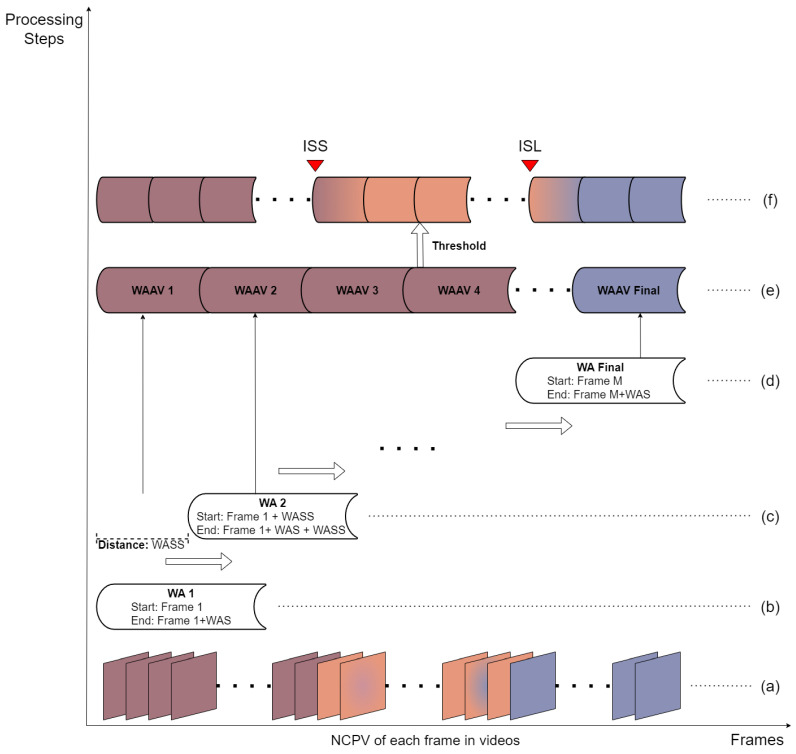
Example of our proposed localization algorithm. (**a**) New composite predict value for each processed frame, obtained from the CPV after K-means clustering. (**b**) First waiting area (WA(1)) and waiting area size. (**c**) Second waiting area (WA(2)) and waiting area slide size. (**d**) Final waiting area (WA(Final)) of the video. (**e**) Waiting area average value (WAAV) for all WAs in a video. WAAV is the key indicator for detecting the intersection of the stomach–small intestine and small intestine–large intestine. (**f**) Intersection of stomach and small intestine (ISS) and intersection of small intestine and large intestine (ISL). Arrows indicate the ISS and ISL.

Figure 4 illustrates the flowchart of our proposed localization algorithm (OPLA). The waiting area (WA) enables the localized analysis of frame sequences without altering the original data. The waiting area slide size (WASS) defines the overlap between consecutive WAs, so that the algorithm can seamlessly transition between adjacent WAs during the subsequent processing. The waiting area average value (WAAV) is the key indicator used by the algorithm to detect the intersections between the stomach–small intestine and small intestine–large intestine, which are determined through threshold processing.

The OPLA algorithm consists of the following steps:

Step 1: The CPV of each frame in the capsule endoscopy video is processed using the K-means algorithm to obtain the NCPV. The purpose of this step is to reduce the noise in the CPV.

Step 2: For the first WA, which spans from the first frame to the (1+WAS)th frame, the new composite predict value (NCPV) of each frame was stored, its average was calculated, and it was saved as WAAV1. The purpose of this step was to smooth the short-term CPV variations.

Step 3: The second WA was created, spanning from frame (1+WASS) to frame (1+ WASS + waiting area size (WAS)) of the video. Following the same process as in Step 2, the average NCPV for this WA was computed and stored as WAAV_2_.

Step 4: Steps 2 and 3 were repeated iteratively until the remaining video frames were insufficient to form a complete WA. This WA was directly deleted from the final incomplete WA.

Step 5: The location algorithm used all the WAAV values to identify the two intersections. First, the stomach–small intestine intersection was located using the small intestine threshold (ST) and the small intestinal continuous threshold (SCT). The intersection of the small intestine–large intestine was determined using the large intestine threshold (LT) and the large intestine continuous threshold (LCT). ST represents the WAAV value at the stomach–small intestine intersection, and SCT confirms that this is a continuous transition and reduces false positives due to short-term fluctuations in WAAV. The principles of the LT and LCT are similar.

Step 6: The time point that marks the stomach–small intestine intersection was defined. The intersection of the stomach and small intestine (ISS) was determined when it satisfied Equations (2) and (3) and its value remained stable.

(2) ISS<ST

(3) $$\frac{1}{SCT}\sum_{i=ISS+1}^{ISS+SCT}i>ST$$

Step 7: The time point that marks the small intestine–large intestine intersection was defined. This intersection of the small and large intestines (ISL) was determined when it satisfied Equations (4) and (5) and its value remained stable.

(4) ISL<LT

(5) $$\frac{1}{LCT}\sum_{i=ISL+1}^{ISL+LCT}i>LT$$

### 2.3. Evaluation Criteria

In this study, precision was used as a metric to evaluate the performance of the image classification model. Precision is defined by Equation (6) as follows:

(6) $$\mathrm{Precision}=\frac{TP}{TP+FP}$$

where true positive (TP) represents correctly classified positive frames, and false positive (FP) represents incorrectly classified frames.

To evaluate the performance of the localization algorithm, the following three metrics were used: (a) the number of error frames (NEF); (b) the mean absolute error frames (MAEF); and (c) the median absolute error frames (MdAEF).

In this study, the errors were quantified by calculating the absolute difference between the algorithm-predicted and endoscopist-determined time points for the gastrointestinal intersections. NEF is calculated using Equation (7) as follows:

(7) NEF=|STP−OPTP|

where STP is the standard time point specified by the endoscopist and OPTP is the time point determined by our proposed algorithm. MAEF is the mean value of NEF across all the videos. MdAEF is the median value of NEF across all the videos.

### 2.4. Implementation Details

The following training hyperparameters were used: learning rate of 1 $\times 10^{-4}$, batch size of 32, and 50 training epochs. These parameters were determined through trials and errors. All experiments, including the training, validation, and testing of the model, were performed on a workstation equipped with an 11th Gen Intel^®^ Core™ i7-11700F CPU (2.5 GHz, 16 cores) and an NVIDIA RTX™ 3080Ti GPU, while the inference speed was evaluated on a workstation equipped with a 13th Gen Intel^®^ Core™ i7-13700F CPU (2.1 GHz, 24 cores) and an NVIDIA RTX™ 4070 GPU.

The proposed network model was implemented using the PyTorch framework (version 1.11). Classification models based on the Swin Transformer, Vision Transformer, ResNet50, DenseNet121, InceptionV4, and VGG19 were also trained and tested using the same data for comparison.

For the K-means algorithm, a box size of 200 with a stride of 200 was used. In the localization algorithm, the WAS parameter was set to 200 and the WASS parameter to 100. The ST and LT were set to 1.35 and 2.4, respectively. The SCT and LCT were set to 8.

## 3. Results

This section presents the classification performance of the different deep learning models on our capsule endoscopy dataset, together with the MAEF and MdAEF for locating the stomach–small intestine and small intestine–large intestine intersections in the different experiments. In addition, ablation studies were conducted to evaluate the effects of the different components of the proposed method on locating the intersection of the alimentary tract. Table 1 lists the settings used in the ablation studies.

### 3.1. Performance of the Proposed Algorithm in the Classification of Images and the Localization of Intersections

Table 2 lists the parameter counts (in millions) and inference speeds (in FPS) of six classification models. Among the CNN-based models, DenseNet121 has the smallest number of parameters, at only 6.96 million, while ResNet50 achieves the fastest inference speed at 585.70 FPS. Among the Transformer-based models, although the Swin Transformer has a relatively small number of parameters, its inference speed is still slower than that of the Vision Transformer, reaching only 184.78 FPS.

Table 3 lists the classification precision, MAEF, and MdAEF obtained with the following four different methods: (a) the classification model alone; (b) the classification model with K-means; (c) the classification model with OPLA; and (d) the classification model with both K-means and OPLA, respectively.

**Figure 5:**
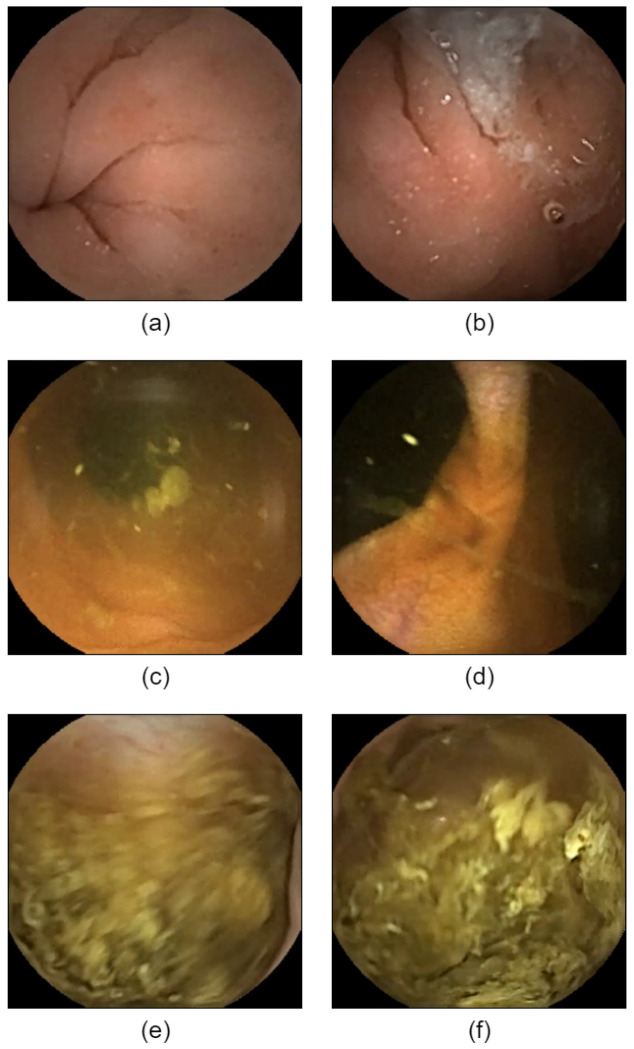
Examples of misclassified frames from capsule endoscopy videos. (**a**) shows a stomach frame incorrectly classified as the small intestine; (**b**) shows a stomach frame misclassified as the large intestine; (**c**) shows a small intestine frame misclassified as the stomach; (**d**) shows a small intestine frame misclassified as the large intestine; (**e**) shows a large intestine frame misclassified as the stomach; and (**f**) shows a large intestine frame misclassified as the small intestine.

ResNet50, DenseNet121, and InceptionV4 achieved the highest accuracies for the stomach (96.79%), small intestine (97.32%), and large intestine (98.93%) classifications, respectively. Of all the classification models used to analyze the capsule endoscopy videos, ResNet50 achieved the lowest MAEF of 77,978 frames. This high MAEF indicates that the use of classification models alone is insufficient for accurate localization. Figure 5a illustrates a stomach frame misclassified as the small intestine; Figure 5b shows a stomach frame misclassified as the large intestine; Figure 5c presents a small intestine frame misclassified as the stomach; Figure 5d displays a small intestine frame misclassified as the large intestine; Figure 5e shows a large intestine frame misclassified as stomach; and Figure 5f presents a large intestine frame misclassified as the small intestine.

The integration of the K-means algorithm with the classification models reduced the MAEF and MdAEF of all the models by an average of 37,590 and 37,494 frames, respectively. In the capsule endoscopy analysis using this method, ResNet50 with K-means achieved the lowest MAEF and MdAEF of 7100 and 502 frames, respectively.

The combination of the Swin Transformer and OPLA achieved the lowest MAEF and MdAEF values of 1026 and 195 frames, respectively. In contrast, DenseNet121 with the OPLA exhibited the highest MAEF of 2806 frames among all classification networks, whereas the combination of a Vision Transformer and OPLA yielded the highest MdAEF of 935 frames. Compared to using the classification network alone or in combination with K-means, the addition of OPLA significantly reduced MAEF and MdAEF. On average, this method reduced the MAEF by 80,629 frames and the MdAEF by 74,553 frames compared to the classification network alone.

The integration of the classification network, K-means algorithm, and OPLA significantly reduced the MAEF and MdAEF. The Swin Transformer, when combined with K-means and OPLA, achieved the lowest MAEF and MdAEF values of 891 and 190 frames, respectively. This corresponds to a reduction of 82,956 frames in the MAEF and 73,758 frames in the MdAEF compared to using the Swin Transformer alone.

### 3.2. Visualization of Alimentary Tract Intersection Points in Capsule Endoscopy

**Figure 6:**
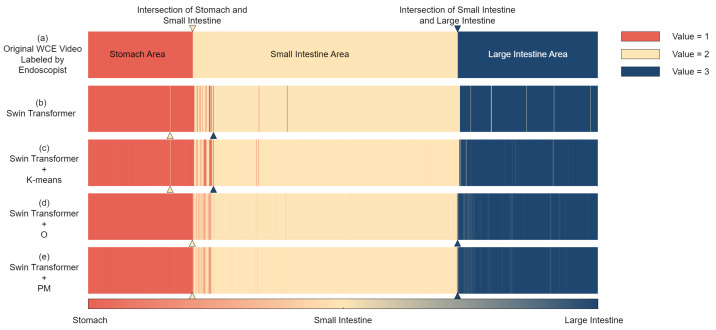
Comparison of the results of localization of organ intersection points in capsule endoscopy videos using different methods. (**a**) Expert annotations from an experienced endoscopist; (**b**) Results from the Swin Transformer model; (**c**) Results combining the Swin Transformer model with the K-means algorithm; (**d**) Results from combining the Swin Transformer model with the OPLA algorithm; (**e**) Results combining the Swin Transformer model with both the K-means and OPLA algorithms. Red, yellow, and blue represent the stomach, small intestine, and large intestine, respectively. Yellow and blue arrows indicate the intersections between stomach and small intestine and small intestine and large intestine intersections, respectively. The gradient color bar at the bottom represents the continuous spectrum of organ Swin Transformer, with values ranging from 1 (stomach) to 3 (large intestine).

The accurate localization of capsule endoscope is crucial for lesion detection and location marking in capsule endoscopy video analysis. A method is proposed based on the Swin Transformer classification model, combined with the K-means and OPLA algorithms, for post-processing the classification results to improve the precision of organ intersection localization. Figure 6 presents a comparison of organ intersection localization results in the capsule endoscopy videos obtained with different methods.

The gradient color bar at the bottom of the image represents a continuous spectrum of organ classifications, with values ranging from 1 to 3. Red, yellow, and blue denote the stomach (1), small intestine (2), and large intestine (3), respectively.

In Figure 6a,b, only solid colors are used to represent the experts’ annotations and the direct outputs of the Swin Transformer model, respectively. Figure 6c–e show the results of the different processing stages, where the color variations reflect subtle differences in the method outputs. This visualization method allows us to intuitively view the results obtained through different processing techniques, which facilitates the identification of organ intersection points and the evaluation of the effectiveness of different methods.

As shown in Figure 6b, using the Swin Transformer model alone leads to some misclassifications, such as misclassifying the stomach as the small or large intestine. The results also showed discrete features and fluctuations, making it difficult to accurately locate the intersections of the alimentary tract. Our proposed method introduces the K-means and OPLA algorithms to postprocess the classification results, effectively improving the precision and clarity in visualizing the localization of the organ intersections.

As the original video contains thousands of frames, when mapped to a single image, some minor classification errors or color gradients may not be fully represented, owing to limitations in the image resolution. To improve the visualization, post-processing was performed on Figure 6b,c to obtain a clearer representation. This procedure helps to demonstrate the effectiveness of the methods while preserving the integrity of the original data.

Figure 6c–e show that these methods progressively improve the clarity of the visualization of the organ intersections. The proposed combination method (see Figure 6e) indicates the time points closest to the expert annotations in Figure 6a, demonstrating its superiority in localizing capsule endoscope. This hybrid approach provides a reliable solution for capsule endoscope localization in capsule endoscopy video analysis.

## 4. Discussion

In this study, we proposed a hybrid method involving a deep neural network-based classifier, a K-means algorithm, and a localization algorithm for the localization of a capsule endoscope in the alimentary tract. The proposed method significantly improved the precision and efficiency of capsule endoscope localization using images as compared to the previous studies. In this study, when ResNet50 was selected as the classification network, we achieved precision values of 96.79%, 97.32%, and 97.81% for the stomach, small intestine, and large intestine classification, respectively. In addition, when the Swin Transformer model was selected as the backbone, MAEF was reduced from 83,847 frames to 891 frames and MdAEF from 73,948 frames to 190 frames. This improvement may be attributed to the ability of the K-means algorithm to effectively cluster the classification results, whereas the OPLA further optimizes the localization of intersection points. Therefore, the proposed algorithm provides the precise time points of alimentary tract intersections using images, thereby reducing interpreting time.

To better understand the factors contributing to misclassification, we analyzed the structural and imaging features associated with different regions of the alimentary tract, as shown in Figure 5 in Section 3. The reasons for misclassification can be summarized as follows: The unique structural features of the large intestine facilitated its recognition. However, the presence of stool in the large intestine may have caused unexpected misclassification, as shown in Figure 5e,f. The classification precision for the small intestine was higher than that for the stomach. The villous structure of the small intestine was more easily recognized by the classification network than the smoother and more variable gastric mucosa. However, the small intestine may be misclassified at its intersection with the stomach and large intestine, where the villous structure is not typical. In contrast, images captured by capsule endoscopy in the stomach may change significantly due to the inner appearance of the stomach, as well as the patient’s posture, making it difficult to extract consistent features thus reducing the classification precision. These findings highlight the critical role of structural and imaging conditions in classification precision, which ultimately impacts the reliability of the localization system.

The limitations of the proposed method are as follows. First, the external validity of this research is not performed because all the data were from a single center. Second, the classification network in our study relied on conventional single-frame classification, where the temporal features in the capsule endoscopy videos were not used. To address these limitations and improve the robustness of the system, future work will include performing a multiple center study to validate the generalization of the proposed method, as well as integrating temporal information into our classification framework, such as combining conventional 2D CNNs and 3D CNNs.

## 5. Conclusions

We proposed a system for localizing the position of a capsule endoscope within the alimentary tract using computer-aided analysis of endoscopic images. Using a Swin Transformer-based classification, we achieved a precision of 97.06% for capsule endoscope images. Using the results from the Swin Transformer model as input, the proposed localization system successfully identified the organ intersections, which were then utilized to locate the capsule endoscope. Experiments on images from 20 participants demonstrated that the transition time errors, calculated by converting 891 frames (485 frames for the stomach to small intestine intersection and 406 frames for the small intestine to large intestine intersection) into a total of 29.7 s (16.2 s and 13.5 s, respectively), were within the clinically acceptable range. These results were obtained from validation videos with an average length of 3,261.8 s. The proposed method demonstrates the potential to assist endoscopists in monitoring the capsule endoscopy process.

## Figures and Tables

**Table 1:** Overview of the ablation setting.

Method	Backbone Network						Post-Processing	
	ST	VT	RN	DN	IV4	VGG	K	OPLA
**Classification Model**								
ST	✓							
VT		✓						
RN			✓					
DN				✓				
IV4					✓			
VGG						✓		
**Classification Model with K-means**								
ST + K	✓						✓	
VT + K		✓					✓	
RN + K			✓				✓	
DN + K				✓			✓	
IV4 + K					✓		✓	
VGG + K						✓	✓	
**Classification Model with OPLA**								
ST + OPLA	✓							✓
VT + OPLA		✓						✓
RN + OPLA			✓					✓
DN + OPLA				✓				✓
IV4 + OPLA					✓			✓
VGG + OPLA						✓		✓
**Classification Model with Proposed Method**								
ST + PM	✓						✓	✓
VT + PM		✓					✓	✓
RN + PM			✓				✓	✓
DN + PM				✓			✓	✓
IV4 + PM					✓		✓	✓
VGG + PM						✓	✓	✓

ST: Swin Transformer, VT: Vision Transformer, RN: ResNet50, DN: DenseNet121, IV4: InceptionV4, VGG: VGG19, CM: Classification Model, K: K-means algorithm, OPLA: Our Proposed Localization Algorithm, PM: Proposed Method.

**Table 2:** Overview of Parameters and Inference Speed.

Model	Swin Transformer	Vision Transformer	ResNet50	DenseNet121	InceptionV4	VGG19
Parameters (Million)	48.84	87.46	23.51	6.96	42.68	139.58
Inference Speed (FPS)	184.78	502.34	585.70	233.50	195.52	279.31

**Table 3:** Performance comparison of different models and methods.

Model	Classification Precision (%)				Stomach to Small Intestine (Frames)		Small Intestine to Large Intestine (Frames)		Total (Frames)	
	Stomach	Small Intestine	Large Intestine	Total	MAEF	MdAEF	MAEF	MdAEF	MAEF	MdAEF
**Swin Transformer**										
ST	93.46	97.28	98.68	97.06	6384	4640	77,463	69,308	83,847	73,948
ST + K	–	–	–	–	3461	355	62,019	60,447	65,480	60,802
ST + OPLA	–	–	–	–	555	85	471	**110**	1026	195
**ST + PM**	–	–	–	–	**485**	**80**	**406**	**110**	**891**	**190**
**Vision Transformer**										
VT	93.79	94.58	97.26	94.80	3635	2276	79,833	75,327	83,468	77,603
VT + K	–	–	–	–	2739	296	55,189	46,306	57,928	46,602
VT + OPLA	–	–	–	–	**734**	**140**	1956	795	2690	935
**VT + PM**	–	–	–	–	758	**140**	**1027**	**360**	**1785**	**500**
**ResNet50**										
RN	**96.79**	**97.32**	97.81	**97.32**	4152	1165	73,826	69,028	77,978	70,193
RN + K	–	–	–	–	2423	162	4677	340	7100	502
RN + OPLA	–	–	–	–	583	**95**	1682	250	2265	345
**RN + PM**	–	–	–	–	**574**	100	**1235**	**110**	**1809**	**210**
**DenseNet121**										
DN	94.21	**97.32**	98.26	97.12	4663	3310	79,921	75,332	84,584	78,642
DN + K	–	–	–	–	3859	186	36,991	27,114	40,850	27,300
DN + OPLA	–	–	–	–	**537**	**100**	2269	**110**	2806	**210**
**DN + PM**	–	–	–	–	541	120	**1883**	**110**	**2424**	230
**InceptionV4**										
IV4	96.56	94.80	**98.93**	95.42	3793	224	79,249	72,979	83,042	73,203
IV4 + K	–	–	–	–	1393	**102**	33,371	23,170	34,764	23,272
IV4 + OPLA	–	–	–	–	570	120	**988**	**125**	**1558**	**245**
**IV4 + PM**	–	–	–	–	**562**	120	998	130	1560	250
**VGG19**										
VGG	95.55	96.92	97.82	96.88	4981	2662	78,685	73,373	83,666	76,035
VGG + K	–	–	–	–	2024	218	62,902	65,965	64,926	66,183
VGG + OPLA	–	–	–	–	594	230	1871	145	2465	375
**VGG + PM**	–	–	–	–	**583**	**190**	**1295**	**135**	**1878**	**325**

ST: Swin Transformer, VT: Vision Transformer, RN: ResNet50, DN: DenseNet121, IV4: InceptionV4, VGG: VGG19; K: K-means algorithm, OPLA: Our Proposed Localization Algorithm, PM: Proposed Method; MAEF: Mean Absolute Error Frames, MdAEF: Median Absolute Error Frames.

## Data Availability

The datasets presented in this article are not readily available because of the restrictions of the IRB. Requests to access the datasets should be directed to zhu.xin@tmd.ac.jp.

## Abbreviations

The following abbreviations are used in this manuscript:

CADX	Computer-aided Analysis
CNN	Convolutional Neural Network
FPS	Frames Per Second
CPV	Composite Predicted Value
OPLA	Our Proposed Localization Algorithm
CP	Classification Probability
NCPV	New Composite Predict Value
WA	Waiting Area
WAS	Waiting Area Size
WASS	Waiting Area Slide Size
WAAV	Waiting Area Average Value
ISS	The Intersection of the Stomach and Small Intestine
ISL	The Intersection of the Small Intestine and Large Intestine
ST	Small Intestine Thresholds
SCT	Small Intestine Continuous Thresholds
LT	Large Intestine Thresholds
LCT	Large Intestine Continuous Thresholds
NEF	Number of Error Frames
MAEF	Mean Absolutely Error Frames
MdAEF	Median Absolutely Error Frames
